# Implementing contact tracing for tuberculosis in Kyrgyz Republic and risk factors for positivity using QuantiFERON-TB Gold *plus*

**DOI:** 10.1186/s12879-020-05465-x

**Published:** 2020-10-12

**Authors:** Caroline Corbett, Aizat Kulzhabaeva, Tatjana Toichkina, Gulmira Kalmambetova, Sevim Ahmedov, Uladzimir Antonenka, Altyn Iskakova, Dilorom Kosimova, Dasha Migunov, Bakyt Myrzaliev, Evgeni Sahalchyk, Nagira Umetalieva, Monica Vogel, Abdylat Kadyrov, Harald Hoffmann

**Affiliations:** 1 Departments SYNLAB Gauting & IML red GmbH, WHO - Supranational Tuberculosis Reference Laboratory Munich-Gauting, Institute of Microbiology and Laboratory Medicine, Robert-Koch-Allee 2, D-82131 Gauting, Germany; 2 fhi360; Defeat-TB program, Bishkek, Kyrgyz Republic; 3 Republican Tuberculosis Reference Laboratory, Bishkek, Kyrgyz Republic; 4 USAID, Washington DC, USA; 5 ABT Associates, Washington DC, USA; 6 KNCV Branch Office in the Kyrgyz Republic, Bishkek, Kyrgyz Republic; 7 Republican Tuberculosis Center, National TB Program, Bishkek, Kyrgyz Republic; 8 SYNLAB Gauting, SYNLAB Human Genetics, Munich, Germany

**Keywords:** Community contacts, Tuberculosis, Latent tuberculosis infection, QuantiFERON, Active case finding, Contact tracing

## Abstract

**Background:**

Effective active case finding (ACF) activities are essential for early identification of new cases of active tuberculosis (TB) and latent TB infection (LTBI). Accurate diagnostics as well as the ability to identify contacts at high risk of infection are essential for ACF, and have not been systematically reported from Central Asia. The objective was to implement a pilot ACF program to determine the prevalence and risk factors for LTBI and active TB among contacts of individuals with TB in Kyrgyz Republic using Quantiferon-TB Gold *plus* (QuantiFERON).

**Methods:**

An enhanced ACF project in the Kyrgyz Republic was implemented in which *close* and household (*home*) contacts of TB patients from the Issyk-Kul Oblast TB Center were visited at home. QuantiFERON and the tuberculin skin test (TST) alongside clinical and bacteriological examination were used to identify LTBI and active TB cases among contacts. The association for QuantiFERON positivity and risk factors were analysed and compared to TST results.

**Results:**

Implementation of ACF with QuantiFERON involved close collaboration with the national sanitary and epidemiological services (SES) and laboratories in the Kyrgyz Republic. From 67 index cases, 296 contacts were enrolled of whom 253 had QuantiFERON or TST results; of those 103 contacts had LTBI (positive TST or IGRA), and four (1.4%) active TB cases were detected. Index case smear microscopy (OR 1.76) and high household density (OR 1.97) were significant risk factors for QuantiFERON positivity for all contacts. When stratified by age, association with smear positivity disappeared for children below 15 years. TST was not associated with any risk factor.

**Conclusions:**

This is the first time that ACF activities have been reported for Central Asia, and provide insight for implementation of effective ACF in the region. These ACF activities using QuantiFERON led to increase in the detection of LTBI and active cases, prior to patients seeking treatment. Household density should be taken into consideration as an important risk factor for the stratification of future ACF activities.

## Background

Tuberculosis (TB) continues to be a major global health challenge with an estimated 10 million people falling ill world wide in 2017, including 1 million children [1]. In an immunocompetent adult the first stage of infection is latent TB infection (LTBI), during which the infected person does not develop clinical symptoms or organic pathology [2]. The World Health Organization (WHO) estimates that about 1.8 billion people are latently infected and that they have a 5–15% lifetime risk of developing TB disease [2].

Active TB is diagnosed based on clinical symptoms, radiological signs and bacteriological confirmation, whereas LTBI is only detectable by indirect immunological tests such as the Tuberculine Skin Test (TST) or interferon-gamma release assays (IGRA) like the Quantiferon-TB Gold *plus* (QuantiFERON) [3–5] or T_SPOT® [6]. Public health authorities use contact investigation and active case finding (ACF) to investigate individuals at risk for acquiring infection from an infectious TB patient (index case) [7–9]. ACF allows for the detection of TB cases prior to individuals seeking treatment, resulting in earlier treatment and decreasing the number of transmission events [7, 9, 10].

The land-locked Kyrgyz Republic is a high TB incidence country (incidence =128/100,000) in Central Asia with one the world’s highest rates of MDR-TB (26% among new cases, and 52% among previously treated cases) [11]. There are currently national guidelines in place to identify household contacts of newly diagnosed index cases to identify LTBI as early as possible, and in the Issyk-Kul region from 2016 to 2018 an average of 309 (range 214–363) contacts were identified. However, there are many weaknesses of the current guidelines that include but are not limited to: ambiguous definitions of who is a contact case or index case, risk groups and assessments are missing from the guidelines, no household visits are performed, and screening is performed with TST in the population with a high BCG vaccination rate (national BCG coverage from 96 to 99% from 2009 to 2019 [12]). It is unclear as to the effectiveness of these guidelines as there are currently no reports or published data on these programs, but it is suspected that contact tracing is not fully implemented. The implementation and reporting of an effective ACF program in Central Asia is essential to reduce TB incidence in this high prevalence country, and use the lessons learned for the implementation in the surrounding regions [13].

Implementing effective ACF with the appropriate diagnostic tools and resources can be challenging, and requires close collaboration between the country’s public TB sector and the local sanitary and epidemiological services (SES). Here, we report on a pilot project of ACF following risk stratified contact investigation utilizing IGRA testing (Quantiferon-TB Gold *plus*) for the first time in the Kyrgyz Republic as a scaffold for the Central Asian Region. Aims of the project were to (i) determine the prevalence of LTBI and active TB among close and household (home) contacts; (ii) to identify predictors of QuantiFERON positivity to inform future risk-stratified contact investigation programs; and iii) to evaluate QuantiFERON and TST results in relation to risk factors.

## Methods

### Index cases

From November 2018 until March 2019, TB patients newly admitted to the Issyk-Kul regional TB Center, Issyk-Kul region, Kyrgyz Republic, were enrolled as index cases when they were (i) over 18 years of age; (ii) independently capable of making decisions; (iii) living in the region for more than 3 months; (iv) diagnosed with bacteriologically confirmed pulmonary TB; and (v) had anti-TB treatment initiated within previous 2 months. Index cases were categorized as either positive (ss+) or negative (ss-) for sputum smear microscopy, and underwent a standardized interview regarding potential contacts and living conditions.

### Contact persons

Individuals the index case reported to have had contact with over the past 3 months prior to hospitalisation were enrolled when they (i) lived in the Issyk-Kul region; (ii) agreed to be visited; (iii) were accompanied by parent or legal guardian if under 18 years old; and (iv) had provided informed written consent. The frequency, intensity and duration of the exposure were key factors in the categorization of contacts. A streamlined approached was employed and contacts were categorized as *home* contacts when living or regularly staying in the same household as the index case (at a minimum 3 months prior to the index case having symptoms or having a presumptive TB diagnosis), or *close* contacts when having had regular contact in closed or poorly ventilated rooms for more than an hour several times per week over the past 3 months prior to the TB diagnosis. For all contact persons, medical conditions fostering TB infection (below 6 years of age, living with HIV, immuno-suppression, renal failure, diabetes) were queried; however, results were only presented to the doctor and not available for analysis. The household risk was assessed as “high” when two or more people on average lived in one room.

### Home visit

A representative of the local branch of the national sanitary and epidemiological services (SES) visited enrolled participants who had home or close contact with the index case and established a history of symptoms (cough, thoracic aches, fever, nigh-sweats, wasting), performed a clinical examination, and for participants over the age of five blood collection for QuantiFERON; for children below the age of six a TST was performed. Symptomatic contacts were presented to local TB physicians who ordered digital chest x-rays in two planes and further TB diagnostics according to local standards. The QuantiFERON result was interpreted following the instructions of the manufacturer (Qiagen, Germany) at the National Reference Laboratory (NRL) in Bishkek. The TSTs were read by the representative of the local SES and assigned “reactive” with indurations of more than 5 mm after 48 to 72 h.

### Sample transport

**Fig. 1:**
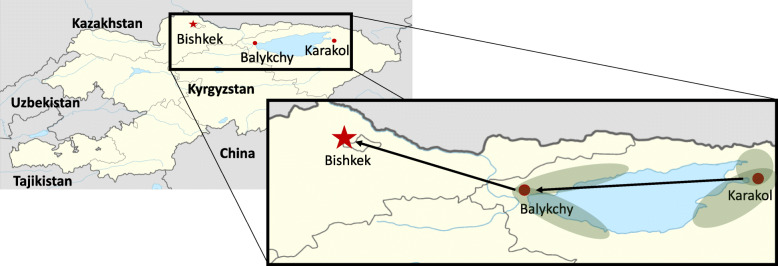
Quantiferon TB Gold *plus* transportation and sampling regions in Kyrgyzstan. Blood samples are collected in the Issyk-Kul region from contacts located in the shaded areas. Samples are transported to the regional hospitals located at either Balykchy or Karakol, before being transported to the National Reference Laboratory in Bishkek

All blood samples for QuantiFERON were transported from the collection site to one of two regional hospitals located in either Balykchy or Karakol within 6 h of collection (Fig. 1). The 16 to 24 h incubation of the test tubes, as per manufacturer instruction, was performed at these hospitals before being collected by the laboratory services AquaLab as per a private public partnership agreement, and transported to the NRL in Bishkek. All following QuantiFERON analyses was performed at the NRL.

### Diagnosis

Active TB was diagnosed based on bacteriological confirmation by doctors in the Kyrgyz Republic who are encouraged to follow the diagnosis algorithm for active TB as recommended by the WHO [14]. LTBI was assigned when the patient was not diagnosed with active TB and had a positive immune response measured by QuantiFERON or by TST (when valid Quantiferon-TB Gold *plus* results were not available).

### Statistical analyses

All statistics were performed using STATA 16.0 (StataCorp LP, College Station, TX, USA). Statistical significance of predictors for either QuantiFERON positivity or symptoms were analysed using logistic regression modelling. Predictors for QuantiFERON positivity included type of contact (home or close), household risk (high or low), presence of symptoms (yes or no), and index case smear microscopy results. Predictors were selected based on the likelihood of transmission from the index case to the contact; household contacts are in close proximity to the index case, household risk indicates density of household living, presence of symptoms has been shown to increase IGRA response, and positive microscopy results of the index case indicate an infectious individual. Two additional logistic regressions were also performed with the outcome defined as either “presence of symptoms” or “TST positive result”. Stratified analysis was separately conducted for adults (≥15 years) and children (< 15 years) due to an association of QuantiFERON positivity with age [15]. Analysis was further stratified by index case smear results [16]. Significant differences were those with a *p* ≤ 0.05.

### Ethical clearance

Permission to conduct the study was obtained from the Ethical Committee of the Kyrgyz Ministry of Health.

## Results

**Fig. 2:**
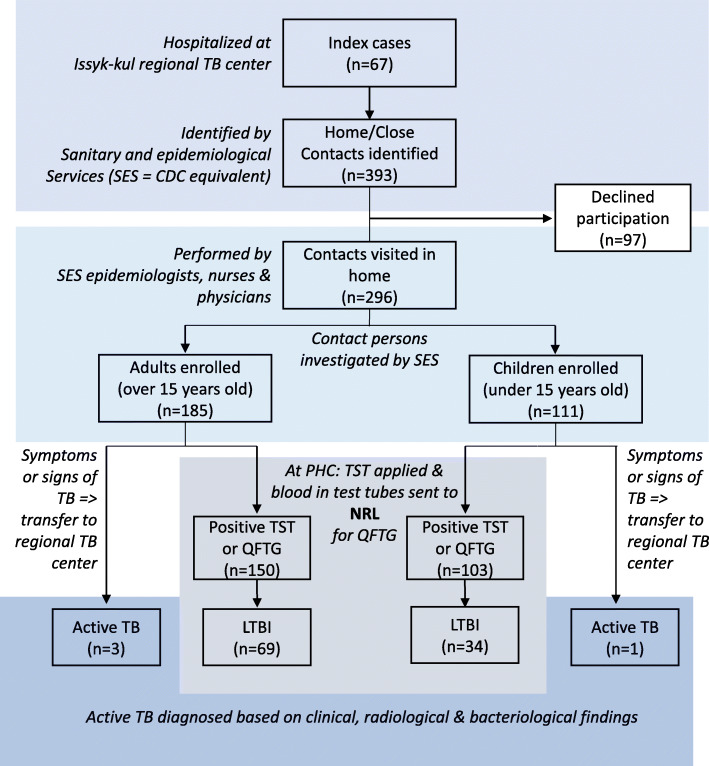
Flow chart representing active case finding methods, locations, institutions, and tests leading to the numbers of patients recruited and diagnosed with latent tuberculosis infection (LTBI) or active tuberculosis (active TB). Locations and institutions are italicized. (Abbreviations: PHC, Primary Healthcare Centre; NRL, National Reference Laboratory)

Sixty-seven index cases were enrolled of whom 35 (52%) were sputum smear microscopy positive. From interviews of the index cases, 393 potential contact persons were identified with 296 (185 adults; 111 children) consenting to enrollment (Fig. 2; Table 1). The majority (210/296, 70.9%) of them were categorized as home contacts (Table 1). Of the 217 contacts that had blood drawn for QuantiFERON testing, three (1.4%) had indeterminate results and were removed from analysis. Thirty-nine contacts (25 children under age six, 13 between six and 14 years of age, and one adult) only had valid TST results for LTBI diagnostics, while 40 contacts had neither QuantiFERON nor TST tested.

**Table 1:** Descriptive characteristics of contacts identified through high priority contact tracing stratified by adults (≥15) and children (< 14)

Category	Combined	Adults (> 15 yrs)	Children (< 14 yrs)
Number of contacts found	296	185	111
Mean age	29.2 yrs.	41.9 yrs.	7.8 yrs.
*range*	*0-87 yrs*	*15-87 yrs*	*0-14 yrs*
Sex ratio (M:F)	0.9	0.85	0.98
IC ss+	144/296	96/185	48/111
	*48.7%*	*52.0%*	*43.2%*
Home contacts	210 /296	122/185	88/111
	*71.0%*	*65.9%*	*79.3%*
Symptoms present	50/296	34/185	16/111
	*16.9%*	*18.4%*	*14.4%*
LTBI^a^	103/253	69/150	34/103
	*40.7%*	*46.0%*	*33.0%*
Active TB^b^	4/296	3/185	1/111
	*1.4%*	*1.6%*	*0.9%*
TB specific changes in chest-x-ray	2/212	2/143	0/69
	*0.9%*	*1.4%*	0.0%
TST positive^c^	35/95	5/7	30/88
	*36.8%*	*71.4%*	*34.1%*
QFTG positive	89/214	69/149	20/65
	*41.6%*	*46.3%*	*30.8%*
TST positive confirmed by QFTG/ TST positive with QFTG result	10/21	2/5	8/16
	*47.6%*	*40%*	*50%*
QFTG positive with IC ss+ / IC ss + with QFTG result	51/105	41/76	10/29
	*48.6%*	*53.9%*	*34.5%*
QFTG positive and active TB / active TB with QFTG result	0/2	0/2	0/0
	*0.0%*	*0.0%*	*0.0%*
QFTG positive and high household risk / high household risk with QFTG result	35/66	26/46	9/20
	*53.0%*	*56.5%*	*45%*
QFTG positive and home contact^d^/ home contact with QFTG result	57/150	41/97	16/53
	*38.0%*	*42.3%*	*30.2%*
QFTG pos & symptoms/ symptoms with QFTG results	19/37	15/27	4/10
	*51.4%*	*55.6%*	*40.0%*
Immune response^e^ & symptoms with chest-x-ray done/Immune response & symptoms	13/22	10/15	3/7
	*59.1%*	*66.7%*	*42.9%*

*Abbreviations*: *IC SS +* index case has positive sputum smear microscopy, *LTBI* Latent Tuberculosis Infection, *TB* tuberculosis, *TST* tuberculin skin test, *QTFG* Quantiferon-TB gold *plus* test

^a^ Latent TB infection: tested for immune response for TB infection (QuantiFERON positive, or TST when QuantiFERON not available) with positive result and not diagnosed as active TB

^b^ Tuberculosis infection: as diagnosed by Doctor based on bacteriological and/or clinical symptoms

^c^ Tuberculin skin test; 5 mm or more considered reactive (positive)

^d^ Home contacts are those who live in the same household as the index case

^e^ Immune response is positive QuantiFERON. When QTFG not available, TST is used

Among the 253 contacts with valid immune test results, 103 (40.7%) individuals with LTBI were identified (Table 1). Of the 22 contact persons having both immune response and symptoms or signs of TB present, 13 (59%) had a chest X-ray performed with one patient having shown changes in the lung typical for TB. For unknown reasons, the local physicians diagnosed this patient having LTBI. Four cases (1.4%; three adults, one child) were diagnosed with active TB (Fig. 2). Of the children under the age of six with TST results (*n* = 25), seven (28%) were reactive.

For all contacts combined, odds of QuantiFERON positivity were increased when index case smear microscopy was positive (OR = 1.76; CI: 1.02–3.06; *p* = 0.043) and when the household risk was high (OR = 1.97; CI = 1.09–3.54; *p* = 0.024) (Table 2). The odds of adults of being positive for QuantiFERON were higher than in children (OR = 1.94; CI = 1.05–3.60; *p* = 0.035). When stratifying by age, adults showed a tendency towards being QuantiFERON positive (OR = 1.88; CI: 0.98–3.61; *p* = 0.057) and had increased odds of presenting with symptoms (OR:2.63, *p* = 0.018, CI: 1.18–5.89), when the index case was smear positive (Table 2). There was no association of being QuantiFERON positive and presenting symptoms or having had home versus close contact. There were no associations of TST and any of the predictors investigated (Table 2).

**Table 2:** Odds ratios for outcomes QuantiFERON-TB Gold *plus* (QFTG) and symptoms with associated risk factors, stratified by age (adults≥15 years of age; children < 15 years of age)

			Combined			Adults			Children		
Outcome	Stratifier	Predictor	OR	95% CI	***p***-value	OR	95% CI	***p***-value	OR	95% CI	***p***-value
QFTG positive	None	Adults vs Children	**1.94**	**1.05–3.60**	**0.035**	–	–	–	–	–	–
		IC ss + vs IC ss-	**1.76**	**1.02–3.06**	0.043*	1.88	0.98–3.61	0.057	1.36	0.48–3.94	0.58
		Symptoms	1.61	0.79–3.29	0.1884	1.57	0.68–3.64	0.289	1.625	0.40–6.54	0.494
		High household risk	**1.97**	**1.09–3.54**	0.024*	1.81	0.90–3.66	0.097	2.53	0.83–7.69	0.102
	Index Case ss+	High household risk	**2.47**	**1.10–5.50**	0.027*	2.16	0.83–5.62	0.115	5.06	0.96–26.66	0.056
TST positive	None	IC ss+	1.75	0.75–4.09	0.195	1.50	0.05–40.63	0.810	1.66	0.68–4.08	0.268
		High household risk	0.85	0.35–2.07	0.720	NA	NA	NA	0.88	0.34–2.28	0.792
Symptoms	None	IC ss+	**1.92**	**1.02–3.58**	0.04*	**2.63**	**1.18–5.89**	0.018*	1.02	0.35–2.98	0.965
	None	High household risk	1.03	0.53–2.01	0.918	1.2	0.53–2.68	0.654	0.76	0.23–2.55	0.655

*Abbreviations*: *IC SS +* index case has positive sputum smear microscopy, *TST* tuberculin skin test, *QTFG* Quantiferon-TB gold *plus* test, *NA* logistic regression not possible as too few results

*Significant at *p* < 0.05; *QFTG* QuantiFERON-TB Gold *plus*, *IC* index case, *ss +* sputum smear microscopy positive

## Discussion

The Quantiferon-TB Gold *plus* has been successfully applied in many contact tracing and ACF projects [3, 5, 15, 17]; however, this is the first time it has been used and reported in an ACF program in Central Asia. The logistics of timely collection, storage, and incubation of blood samples from rural areas as far as 400 km from the place of analysis are a few of the challenges that are overcome with close collaboration and communication between the country’s public TB sector, local SES and private public partnerships established with laboratories in the region. This is the first time an ACF program in the Central Asian region has been reported, and the information is beneficial for implementing future ACF programs in surrounding countries. Additionally, QuantiFERON should be considered for the detection of LTBI in these high-prevalence regions where vaccination is common and cross-reaction with TST is highly likely.

During the 5 months of this pilot ACF program, 393 contacts were identified of which 296 consented to enrollment demonstrating a marked increase from the national program. LTBI was diagnosed in 41% of screened contacts (*n* = 253), which was within the ranges reported in a recently conducted meta-analysis in low-middle income countries [18]. Although we did not see a difference in the proportions of reactivity in QuantiFERON versus TST (39.7% versus 36.8%; respectively), QuantiFERON positivity was associated with previously known risk factors such a smear microscopy of the index case and household density, whereas TST was not. The lack of association with TST most likely resulted from its lack of specificity due to the systematic BCG vaccination in Kyrgyz Republic [14]. Therefore, QuantiFERON is likely better than TST at identifying LTBI cases [4] who would benefit from treatment which would prevent TB re-activation.

The upcoming WHO recommendations for programmatic management of TB preventive treatment and United Nations General Assmebly targets for LTBI treatment of adult contacts require an urgent upscaling of active case finding and LTBI detection [14]. This pilot study indicates that IGRA are a feasible method that can be implemented in high-burden settings like the Kyrgyz Republic, and similar countries in Central Asia. Currently the WHO recommendations do not require TST or IGRA testing before initiation of LTBI treatment; however, testing will be essential since the LTBI prevalence among contacts is not 100%. It should also be taken into consideration that in the Kyrgyz Republic, 1 in 3 infected individuals have MDR-TB. Accurate identification of these infected individuals and LTBI contacts is essential for preventative treatment as recommended by WHO guidelines [14].

In the current study, as in previous studies, smear positivity of the index case was a strong risk factor for LTBI for all contacts combined [8, 10, 16]. However, when stratifying by age, this association completely dissipated for contacts below 15 years of age suggesting that children are also at high risk to be infected by smear negative cases. Particularly for children, living in a densely populated household was a much stronger risk factor for TB infection than the smear status of the index case, as finding that is supported in the literature [3, 8, 15, 19]. Currently, WHO defines only household contacts per se as high risk [14], but our findings suggest that the average household density should be considered as criterion for risk-stratified contact investigation among children in Kyrgyz Republic.

In our ACF pilot project, active TB was diagnosed in 1.4% of screened contacts, a proportion below the average of 3.1% reported in a systematic review [18]. However, as we were not able to follow-up on suspects, diagnosis of active TB heavily relied on rapid bacteriological confirmation. In the same review, the proportion of bacteriologically confirmed TB was 1.2% which is fully in line with our findings [18]. Furthermore, it is likely that active TB was under-diagnosed in the current study despite TB diagnostics being integrated in the regular algorithm following national standards. Chest x-rays were only ordered for about half of patients with both symptoms/signs and immune responses, despite it being a key diagnostic tool for TB-suspects [20]. One patient was not diagnosed as active TB although he had a positive QuantiFERON, TB-typical symptoms and signs as well as changes in the chest-x-ray. This may indicate the need of more intensive training of TB doctors particularly for decision making based on QuantiFERON results which has so far not been available in the Central Asian region. It is likely that more active TB cases would have been identified if the diagnostic algorithm for active TB had been followed, and QuantiFERON positive contacts were followed-up for a period of at least 1 year.

## Conclusions

In conclusion, this is the first time ACF using IGRA has been reported on in Central Asia, leading to the identification of LTBI and active TB in the Kyrgyz Republic. Densely populated households should be considered as an equally important risk factor for TB infection among children as smear positive index cases currently considered in ACF programs. Identification of LTBI cases is necessary to ensure access to preventative treatment leading to shorter regemins, compliance with treatment and prevention of reactivation. As this is a high prevalence country where 1 in 3 TB patients suffer from an MDR-TB infection, this pilot ACT project provides a scaffold for implementation and risk stratification of future programs in surrounding countries in Central Asia with similar TB environments.

## Data Availability

All data generated or analysed during this study are included in this published article.

## Abbreviations

ACF: Active case finding
IGRA: Interferon-gamma release assays
LTBI: Latent tuberculosis infection
MDR-TB: Multi-drug resistant tuberculosis
NRL: National Reference Laboratory
OR: Odds ratio
SES: Sanitary and Epidemiological Services
TB: Tuberculosis
TST: Tuberculin skin test
WHO: World Health Organization
